# High Data Output and Automated 3D Correlative Light–Electron Microscopy Method

**DOI:** 10.1111/j.1600-0854.2008.00815.x

**Published:** 2008-09-16

**Authors:** Giuseppe Vicidomini, Maria C Gagliani, Michela Canfora, Katia Cortese, Fabio Frosi, Clara Santangelo, Pier Paolo Di Fiore, Patrizia Boccacci, Alberto Diaspro, Carlo Tacchetti

**Affiliations:** 1 Centro di Ricerca MicroSCoBiO, Università di Genova16132 Genoa, Italy; 2 LAMBS-Dipartimento di Fisica, Università di Genova16132 Genoa, Italy; 3 Dipartimento di Informatica, Università di Genova16132 Genoa, Italy; 4 IFOM, Fondazione Istituto FIRC di Oncologia Molecolare20139 Milan, Italy; 5 Dipartimento di Medicina Sperimentale, Università di Genova16132 Genoa, Italy; 6 Dipartimento di Medicina, Chirurgia ed Odontoiatria, Universita’ di Milano20142 Milan, Italy; 7 Istituto Europeo di Oncologia20141 Milan, Italy

**Keywords:** confocal microscopy, correlative microscopy, cryosection, electron microscopy, immunofluorescence, immunogold, morphometry

## Abstract

Correlative light/electron microscopy (CLEM) allows the simultaneous observation of a given subcellular structure by fluorescence light microscopy (FLM) and electron microscopy. The use of this approach is becoming increasingly frequent in cell biology. In this study, we report on a new high data output CLEM method based on the use of cryosections. We successfully applied the method to analyze the structure of rough and smooth Russell bodies used as model systems. The major advantages of our method are (i) the possibility to correlate several hundreds of events at the same time, (ii) the possibility to perform three-dimensional (3D) correlation, (iii) the possibility to immunolabel both endogenous and recombinantly expressed proteins at the same time and (iv) the possibility to combine the high data analysis capability of FLM with the high precision–accuracy of transmission electron microscopy in a CLEM hybrid morphometry analysis. We have identified and optimized critical steps in sample preparation, defined routines for sample analysis and retracing of regions of interest, developed software for semi/fully automatic 3D reconstruction and defined preliminary conditions for an hybrid light/electron microscopy morphometry approach.

In recent years, fluorescence light microscopy (FLM) has moved beyond the qualitative viewing of labeled objects. Improved optical and laser equipments, fluorescence-based labeling methods, computer technology and image analysis software have made possible the study of the location, orientation and dynamics of specific biomolecular structures from organelles to single particles (1). Despite the success and wide application (2) of wide-field microscopy (WFM), two-photon excitation microscopy and confocal laser scanning microscopy (CLSM), some limitations still remain. In particular, for typical wavelengths in the visible range, CLSM optimally resolves 180 nm in the focal plane (*x,y*) and 500–800 nm along the optical axis (*z*). In addition, fluorescence microscopy is unable to identify relationships between labeled and unlabeled cellular compartments.

Electron microscopy (EM) and immunoEM can complement FLM analysis because of their higher resolution and ability to visualize labeled and unlabeled cellular compartments. A number of correlative light/EM microscopy (CLEM) methods have been developed to fill the FLM–EM gap (3–11). CLEM can be ‘ideally’ defined as an imaging platform able to capture the same objects by means of multimodal microscopy techniques. This allows for deeper insights in the true size, shape and interconnections of subcellular structures as well as precise mapping of antigen localization. Coupling a dynamic (FLM) to a high-resolution (transmission electron microscopy, TEM) approach, CLEM methods can also add temporal dimensions to the analysis of *in vivo*events (3–6,12–14). Notwithstanding the overall potentiality, CLEM methods still exhibit a number of problems. Among these are the low rate of data output, the low label sensitivity and specificity, the difficulty to perform simultaneous multiple labeling of endogenous and recombinantly expressed antigens or to correlate three-dimensional (3D) FLM with 3D TEM images (15).

We have designed and realized a novel CLEM approach (high data output CLEM, HDO-CLEM), able to potentially perform CLEM analysis on hundreds of events per study session. Our method allows the simultaneous labeling of both recombinantly tagged and endogenous antigens, at the FLM and TEM level, and to obtain the correlation of 3D FLM with 3D TEM images (3D CLEM). In this study, we provide the complete description and validation of the HDO-CLEM ‘package’ that includes process architecture, with defined sample preparation/labeling protocols and dedicated software for image analysis and 3D modeling.

## Results

### Model system

**Figure 1:**
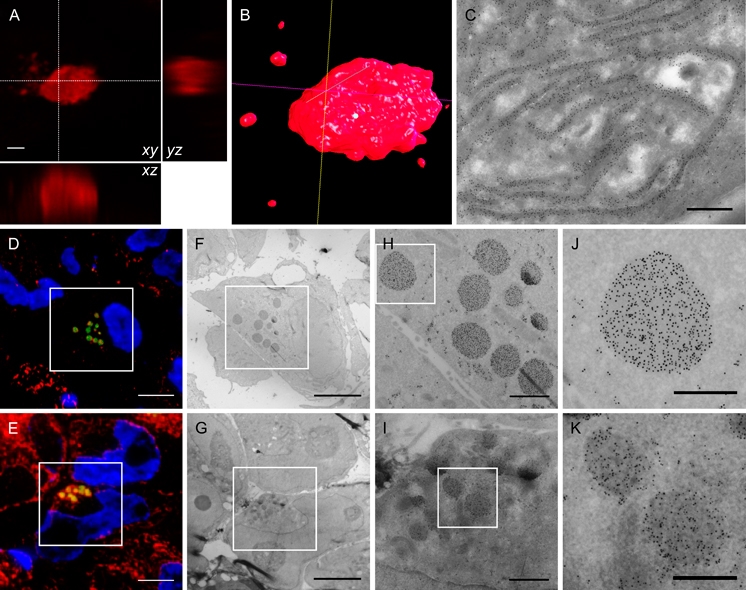
Ultrathin cryosections: good morphology and antigen preservation at FLM and EM level SRB (A–C)- and RRB (D–K)-expressing HeLa cells. A) Immunofluorescence labeling for μ-ΔCh1 (Cy3). CLSM optical sectioning poorly solves the complex tubular architecture of a SRB imaged through the *xy*middle plane of a 3D stack; *xz*axial views along the dotted lines are shown. B) Transparent surface rendering of the 3D stack shown in (A), obtained by MicroSCoBiOJ software. The 3D organization of the SRB tubular structures is not identifiable. The white dot in (B) corresponds to the cross-point of the dotted lines in (A). C) Immunogold labeling for μ-ΔCh1. TEM image of a 200 nm thick cryosection of a SRB showing the intricate tubular structure. D–K) Double immunolabeling on 60 nm (D, F, H and J) or 200 nm (E, G, I and K) cryosections of RRBs. D and E) Immunofluorescence colocalization of calreticulin (Cy3, red) and μ-ΔCh1 (Cy2, green). Nuclei labeled with DAPI (blue). Images were collected either by CLSM (D) or by WFM (E). F–K) Immunogold labeling colocalization of calreticulin (10 nm) and μ-ΔCh1 (15 nm) of the same sections shown in (D and E). H–K) Higher magnification of the squared areas in (F and G) and (H and I), respectively. Higher magnification views of larger areas of the EM images displayed here are shown in Figure S2A–B and ETM Microscopy analysis in Video S1. Bars: 2 μm (A), 1 μm (C), 5 μm (D–G), 1 μm (H and I) and 0.5 μm (J and K).

Rough endoplasmic reticulum (RER) and ER–Golgi intermediate compartment (ERGIC) (16) display a tubular network and assume diverse morphologies under specific conditions, such as increased protein storage (17). We have previously shown (17,18) that HeLa cells stably transfected with a mouse immunoglobulin μ chain, lacking the first constant domain (μ-ΔCh1), together with a wild-type immunoglobulin λ light chain (λ), display spherical dilated RER cisternae resembling the Russell bodies identified in plasmocitoma cells (rough Russell bodies, RRBs). RRBs are about 1 μm in diameter and contain protein aggregates of mutant immunoglobulin M (17,18). Conversely, when μ-ΔCh1 is transfected in the absence of λ, it aggregates in the smooth ERGIC tubular compartments (smooth Russell bodies, SRBs), as shown by the association with the ERGIC marker ERGIC-53 (17). SRBs are composed of narrow (∼100 nm wide, n-SRB) or dilated (∼200 nm wide, d-SRB), curled, tubular cisternae. When analyzed by 3D reconstruction of optical CLSM sections, SRBs, identified by immunofluorescence labeling of μ, showed tightly packed and fused tubular structures. TEM analysis recognized instead well-separated individual tubular structures (Figure 1A–C). The SRB morphological differences between CLSM and TEM are likely because of imaging artifacts linked to the low *z*-axis resolution of the CLSM.

### Specimen preparation protocol and immunolabeling

We based our CLEM method on the use of ultrathin cryosections. Ultrathin cryosections, pioneered by Tokuyasu (19), widely used in combination with immunogold labeling (20), had already proven to be the method of choice for multiple TEM immunolabeling of endogenous and recombinantly expressed antigens and an excellent substrate for a highly detailed immunofluorescence microscopy (8,10,21).

Cryosections were cut randomly through a pellet of RRB- or SRB-expressing HeLa cells. Sixty or 200 nm thick sections, used for detailed two-dimensional (2D) or 3D analysis, respectively, were placed on gold finder grids and used for both FLM and TEM observations (Figure 1D–K). We labeled μ-ΔCh1 by immunofluorescence and immunogold on the same section either alone or in combination with the ER marker calreticulin (22). CY3- and CY2-labeled second-step antibodies and protein A-gold (PAG) were used as tracer for FLM and TEM, respectively (Figure 1). To reduce reflection background and spherical aberration artifacts during FLM observation, finder grids were mounted between a microscope slide and a coverslip. Application of a layer of methylcellulose on the grids guaranteed their prolonged and repeated use for FLM imaging, without significant fluorescence loss, avoiding potential damage during recovery for TEM analysis. Unexpectedly, the methylcellulose film greatly reduced the fluorescence photobleaching, making superfluous the use of anti-fading agents. The methylcellulose coat was washed out before TEM observation to allow for EM staining. Additional examples of labeling for recycling compartments, late endosomes/lysosomes and Golgi apparatus are shown in Figure S1.

### FLM imaging: routine procedure for region of interest identification

**Figure 2:**
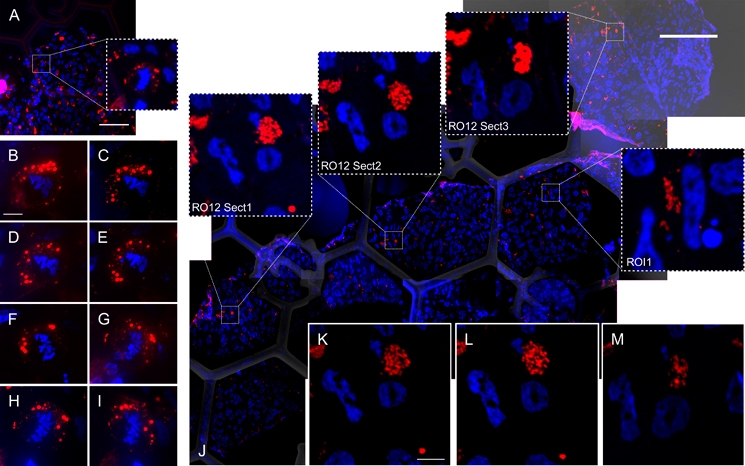
ROI identification at the FLM analysis Immunofluorescence labeling for μ-ΔCh1 (Cy3) on (A–I) RRB- or (J–M) SRB-expressing HeLa cells; nuclei labeled with DAPI (blue). A) A specific ROI is identified by the ‘conventional’ approach (i.e. an M-phase cell containing RRBs); inset represents digital enlarged image of the ROI boxed in (A). B–I) WFM high-magnification imaging of the ROI in (A), identified in eight consecutive serial sections (H corresponds to inset in A). J) ‘Work-on-map’ approach. High-resolution CLSM map of three consecutive serial cryosections. Automatic MosaicJ montage of 16 multichannel images (DAPI-Cy3-Bright field). Bright field channel the grid mesh bars and letters. Images acquired with the maximum image size allowed by the CLSM system (2048 × 2048 pixels, 100 nm pixel size). Insets (ROI1, ROI2 Sect1, ROI2 Sect2 and ROI2 Sect3) are direct digital enlargements of the corresponding areas boxed in (J). ROI2 Sect1–3 show the same ROI identified on three consecutive serial sections. K–M) Manual image acquisition of ROI2 Sect1–3 (50 nm pixel size). Bars: 50 μm (A), 5 μm (B), 100 μm (J) and 5 μm (K).

To identify the highest number of region of interest (ROI) on a cryosection, we acquired FLM maps of the fields of interest. As proof of principle, we systematically screened the grids by the eyepiece (‘conventional’ approach) to generate a low-magnification (×20 objective) fluorescence map of selected groups of ROIs (Figure 2A). A subsequent high-magnification/high-resolution imaging (×100 objective) of each individual ROI was then recorded for the FLM/TEM correlation analysis (Figure 2B–I).

Subsequently, we designed an optimized ‘work-on-map’ procedure to increase the efficiency of FLM ROI identification and retracing at the TEM. Spatially consecutive high-magnification images (×60 objective) of single, or serial, sections were automatically acquired and merged by the ‘MosaicJ’ software (23) into a single high-resolution fluorescence map (Figure 2J). The automated work-on-map high-resolution procedure greatly shortened the time needed for image acquisition. Figure 2J shows a CLSM montage of an area including three serial cryosections and containing several potential ROIs (i.e. about 100 cells/grid mesh). Finder grid reference points, that is mesh bars and mesh letters, were included in the map to identify ROI positions (Figure 2J). Mesh position and nuclei, or RBs, patterns were essential to retrace selected ROIs at the TEM. Further FLM acquisition steps were decisive in the case of signal saturation, instability of focus position, poor pixel sampling or chromatic aberration problems in the initial imaging (Figure 2K–M).

### FLM imaging: 3D volume rendering

Fluorescence light optical sectioning microscopy techniques (24) and in particular CLSM (24) are common approach to 3D analysis of biological samples. The 3D modeling obtained from optical sectioning helps to define the spatial organization of subcellular organelles inside the sample. However, the relative poor axial resolution of CLSM, because of out-of-focus fluorescence background, limits the definition of subcellular compartments. CLEM approach, combined with the use of serial 200 nm cryosections, can solve this problem because the thickness below the resolution of the CLSM reduces the out-of-focus background, improving the fidelity of the 3D FLM modeling (Figure S3).

We used the automated work-on-map approach to track down corresponding ROIs on ribbons of three to eight consecutive serial cryosections, laid on a single finder grid. The pattern of cell nuclei, or RBs, allowed an *a posteriori*manual screening of the montage to identify the same ROI in serial sections. The 3D surface rendering from physical sections required a registration (e.g. aligning) process. For this reason, we developed a user-friendly software for registering series of bidimensional multichannel images. Registration (25) and rendering software were based on ImageJ(26) plug-ins, either modified or originally developed, and collected together in a software package named MicroSCoBiOJ.

**Figure 3:**
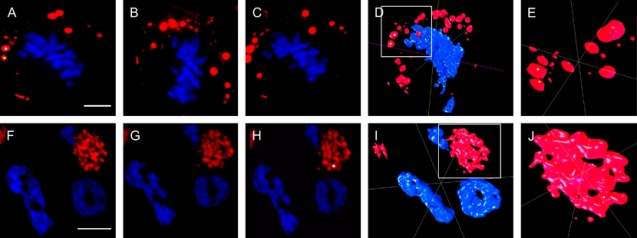
Registration and surface rendering of serial RB FLM images Immunofluorescence labeling for μ-ΔCh1 (Cy3) on (A–E) RRB- or (F–J) SRB-expressing HeLa cells; nuclei labeled with DAPI (blue). A, B, F and G) ROIs (RRBs and SRB, respectively) identified on consecutive 200 nm serial cryosections. Images are selected from Figure 2. C and H) Warped images obtained by affine transformation using defined landmarks. C) Registration of (A, target image) on (B, source image) using chromosomes (blue channel, DAPI), followed by registration of RRBs (red channel). H) Registration of (F, target image) on (G, source image) using SRB (red channel, Cy3) to fix landmarks. D) Surface rendering of the final registered 3D stack from the eight consecutive serial sections displayed in Figure 2B–I by Turboreg MicroSCoBiOJ iteration of the process used to obtain C). I) Surface rendering of the final registered 3D stack from the three consecutive serial sections displayed in Figure 2K–M by Turboreg MicroSCoBiOJ iteration of the process described to obtain (H). E and J) Higher magnification of details boxed in (D) and (I), respectively. The white and yellow dots in (D and E) and the white dot in (J) locate areas in the 3D rendering, previously identified in (A) and (H), respectively. 3D movies of (D–I) are available in Videos S2 and S3. Bars: 5 μm.

Registration was performed between consecutive pair of images (a reference image and a source image). We set the ROI identified on the first section as reference image (Figure 3A,F) and the corresponding ROI on the following section as the source image (Figure 3B,G). For each consecutive pair of serial images, the software automatically iterated the process. Nuclei, or RBs, pattern was used as reference to fix landmarks and to register the respective channel of consecutive serial section images (Figure 3A–C,F–H). The software automatically refined landmarks to minimize the mean square differences between the target image and the warped image (25). These landmarks were successively used to register other objects displayed on separate channels, within the same ROI. When occasional dramatic differences in reference patterns occurred between consecutive cryosections, a manual mode of registration was preferred, disengaging the automatic refinement procedure. Surface rendering and navigation functions embedded in MicroSCoBiOJ originated the final 3D modeling of the ROI and the rotation along different axis (Figure 3D,E,I,J and Videos S2 and S3). As expected, 3D reconstruction from physical sectioning leads to a substantial increase in axial resolution of FLM (8) (Figure S3). As a result of the 3D reconstruction, we identified connections occurring between portions of the tubular network of SRBs, otherwise not identifiable by optical sectioning or analysis of single cryosections (Figure S4).

### Correlation analysis: retracing and superimposition of ROI at TEM level

To efficiently retrace at the TEM level the ROIs identified at the FLM, we generated composite low-magnification TEM map images employing the EM compustage and the automated functions embedded in the EM camera software.

**Figure 4:**
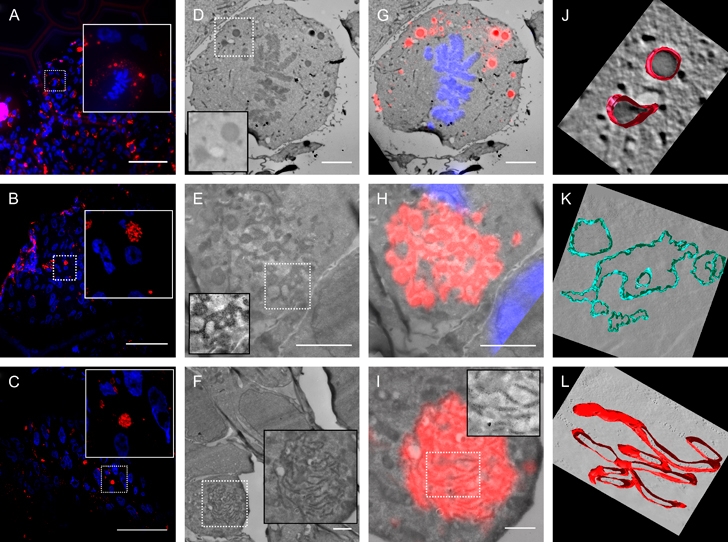
CLEM analysis: retracing ROIs at the TEM A–C) Immunofluorescence labeling for μ-ΔCh1 (Cy3) on RRB (A)-, d-SRB (B)- or n-SRB (C)-expressing HeLa cells (same as in Figure 2A,H,J, respectively); nuclei labeled with DAPI (blue). Solid line insets display higher magnification/resolution views of areas in dashed boxes. D–F) TEM images of (A–C), respectively. Black line inset shows higher magnification of areas encircled in the white line boxes and highlight two selected RRBs (D) and portion of a d-SRB and a n-SRB (E and F, respectively). G–I) CLEM overlay of WFM (D) and CLSM (H and I) on the corresponding TEM images. J–L) ETM analysis of the areas displayed in the insets in (D, E and I) (Videos S4–S9). Virtual sections of the tomogram are set as background images. ETM obtained from a −45° to a +45° tilt series, with 1° increments. Voxel sizes: (J) 2.1 × 2.1 × 2.1 nm (1024 × 1024 × 54 voxels), (K) 22.7 × 22.7 × 7 nm (240 × 159 × 28 voxels) (original tomograph was interpolated to obtain a better 3D model), (L) 1.5 × 1.5 × 1.5 nm (1024 × 1024 × 41 voxels). Bars: 50 μm (A–C), 5 μm (D and G), 2.5 μm (E and H), 2 μm (F) and 1 μm (I).

Grid mesh landmarks and nuclei patterns allowed an easy recognition of the ROI at the TEM level. TEM and FLM spatial ROI map overlay enabled the correlation of ROIs. This operation required resampling and registration of FLM images with TEM images as target (Figure 4A–I). FLM/TEM image superimposition and correlation showed that CLSM was not able to completely resolve the tubular shape of SRB structures. This was mostly because of the limited resolution of the CLSM compared with TEM (Figure 4G–I and Table S1).

However, because TEM images are 2D superimposed projections of structures located at different *z*-levels within the cryosection thickness, they are still unable to fully resolve the complete 3D tubular architecture (Figure S3). For an improved analysis, we performed electron tomography (ETM) of selected ROI areas. The same RBs were first analyzed by FLM 3D reconstruction. Subsequently, any given section of the series was further analyzed by 3D TEM modeling at nanometer resolution (Figure 4J–L and Videos S4–S9). As predicted (Figure S3), the correlation of the 3D FLM with 3D ETM models (3D CLEM) revealed finer details of tubular connections (Figure 4L). Of note, the HDO-CLEM analysis was also successfully applied to cells expressing green fluorescent protein (GFP)-tagged proteins (Figure S5). An approximate timetable of all possible steps of HDO-CLEM is shown in Table S2.

### Correlation analysis: HDO-CLEM morphometry

Morphometry analysis of biological samples can lead to quantitative geometrical information (e.g. surface and volume) and/or antigen density. While FLM provides high-throughput morphometry, with poor accuracy, TEM provides accurate morphometry with low efficiency. FLM and TEM morphometry analysis on the same structures can lead to a potential systematic error between the two approaches. This systematic error could potentially be used to optimize and/or calibrate FLM morphometry analysis, setting the bases for a high-throughput, high-resolution, hybrid FLM/TEM morphometry method.

**Figure 5:**
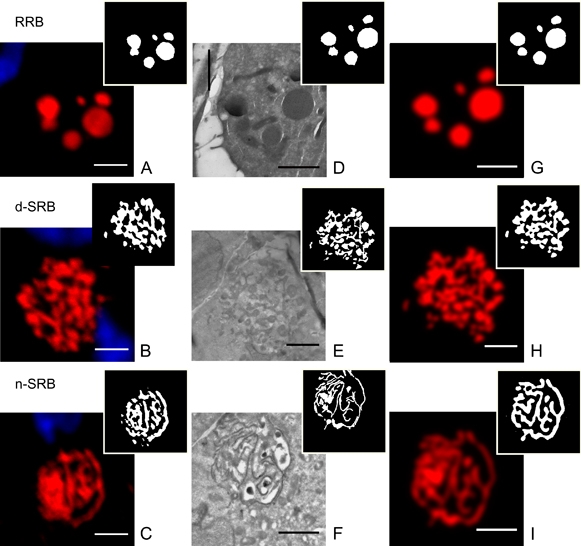
HDO-CLEM morphometry analysis A–C) CLSM images of RRBs, (A) d-SRB (B) and n-SRB (C), immunofluorescence labeled for μ-ΔCh1 (Cy3). Insets, segmented images obtained by WAT_MicroSCoBiOJ. D–F) TEM images of the RBs shown in (A–C). Insets represent the segmented images (manual membrane contouring). G–I) CLSM simulation images of (D–F) obtained convolving segmented TEM images with a point spread function (see Image process formation in File S1). Simulation takes into account all imaging conditions used for the original CLSM imaging in (A–C). Insets, segmented images obtained by WAT MicroSCoBiOJ. Bars: 2 μm.

**Figure 6:**
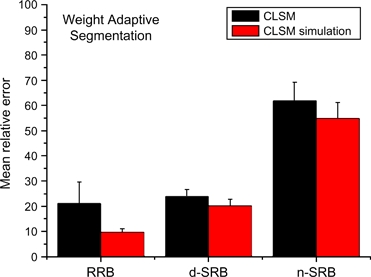
Weight adaptive segmentation Reference values obtained from TEM images (Figure 5D–F) are used to monitor the fidelity of the CLSM measurements for each structure, computing the relative error. Graph displays the mean relative error for the three different morphologies of RBs shown in Figure 5A–I obtained by real (black bars) and simulated (red bars) CLSM images.

To validate such an approach, we selected the three different morphologies of RBs, that is RRBs, d-SRBs and n-SRBs, and performed CLSM imaging (Figure 5A–C). Each RB structure was imaged by EM (Figure 5D–F), and a manual membrane contour was obtained for each image (insets in Figure 5D–F). An automatic segmentation plug-in, developed in our laboratory (weight adaptive threshold MicroSCoBiOJ,WAT-MicroSCoBiOJ), embedded in the MicroSCoBiOJ package (see Segmentation in File S1), was applied to CLSM images (insets in Figure 5A–C) of the same RBs. Figure 6 shows the fidelity of the CLSM surface measurements compared with the ‘real’ TEM measurements. The low mean relative error statistics (RRB 32 ± 6 SEM, d-SRB 37 ± 5 SEM and n-SRB 79 ± 4 SEM; RRB *n*= 8, d-SRB *n*= 5 and n-SRB *n*= 7) indicated the systematic nature of the relative error distribution. These data demonstrated that the major source of the systematic error was because of blurring (related to the band-limited nature of the CLSM) and, as expected, that it decreased for comparatively bigger structures. We next convoluted the three different models of RB, with a theoretical CLSM point spread function (see Image process formation in File S1), to simulate the image process formation (Figure 5G–I). Comparison between the real and the simulated images provided validation to our approach (Figure 6 and Figure S6). CLSM and CLSM simulation relative errors showed a similar behavior for the subresolved d-SRB and n-SRB structures, indicating that they are mostly because of the lower spatial resolution and the blurring introduced by the CLSM imaging. On the contrary, for the non subresolved RRBs, the discrepancy observed between CLSM and CLSM simulation errors indicated that the segmentation algorithm, used for the automatic segmentation of CLSM images to measure the surface of the RB structures, is the major source of the systematic error.

Knowledge of the systematic error allowed defining the fidelity of the FLM morphometry. Therefore, morphometry analysis can be performed on a large scale of samples by CLSM and analyzed with the accuracy of TEM analysis by correcting for the calculated systematic error.

## Discussion

CLEM has proven to be a powerful approach to fill the gap between FLM and TEM imaging, providing greater insight into biological structures and their dynamic interconnections and a precise spatial information of protein localization. Several promising CLEM methods have been developed in the recent years; however, they still exhibit a number of problems and are not used on a routine basis by standard equipped imaging laboratories. Current CLEM, applied on tissue culture cells imaging, use plastic-embedded samples and either are performed on single cells (3), penalizing the possibility to obtain statistically relevant data, or are not suitable for multiple labeling of nonrecombinantly tagged antigens (5,7,12) or 3D analysis.

We have developed a robust CLEM ‘package’ (HDO-CLEM) matching the following requirements: (i) high data output rate, (ii) multiple labeling of both endogenous and recombinantly expressed antigens, (iii) 3D analysis of the structures of interest and (iv) affordability by many TEM laboratories. HDO-CLEM package includes the specific protocols for sample preparation/labeling and a complete software toolbox (MicroSCoBiOJ) for image analysis and 3D reconstruction.

Although several very promising hybrid FLM/TEM labels, able to penetrate an intact cell, have been developed in the recent years [e.g. fluorescence photo-oxidation of diaminobenzidine (5,12) and quantum dots (4,6)], we have pointed to the physical sectioning of the sample, as a solution to antigen accessibility. The use of ultrathin cryosections, pioneered by Tokuyasu (19) and optimized mostly thanks to the founding work of Slot and Geuze (20), is an established method of choice for TEM immunolabeling, with respect to membrane visibility, preservation and displaying of ultrastructural details, maintenance of immunoreactivity and antibody penetration. In particular, excellent immunogold procedures to label cryosections have been developed (20). Ultrathin cryosections serve also as an excellent substrate for high-resolution immunofluorescence microscopy, providing several advantages, that is sharper images, higher *z*-axis resolution and lower fluorescence background (8,10,21). Finally, cryosections do not suffer from the sample shrinkage because of the dehydration procedures used for plastic embedding and are suitable for 3D analysis both at the FLM and at the TEM level. Consequently, we could develop MicroSCoBiOJ software and set conditions for a rapid HDO-CLEM 3D analysis.

The are at least three relevant aspects about the use of HDO-CLEM that make this method novel and possibly able to overcome some limits of other CLEM approaches.

The first is the combination of (i) the well-known suitability of ultrathin cryosections for immunogold and immunofluorescence labeling to obtain images of multiple endogenous and recombinant antigens, identified by both labeling methods, on the same section and (ii) the original use of automated composition of large field maps of cells, both at the light and at the electron microscope level to allow for an extensive subsequent correlation of the images. The method has been applied successfully using both standard fluorochromes and/or GFP-tagged proteins for immunofluorescence, showing a certain versatility of use, and solving some of the problems encountered by the use of other hybrid-labeling agents for CLEM.

In particular, quantum dots fluorescence is quenched by heavy metal staining so that CLEM has to be performed on samples with suboptimal ultrastructural preservation because of the omission of glutaraldehyde fixation and osmium postfixation (4). Fluorescence photo-oxidation of diaminobenzidine, instead, allows the labeling of recombinantly expressed proteins but not of endogenous antigens (5,12). Finally, fluoronanogolds, an alternative FLM/TEM labeling method for cryosections (19,27), rely on silver enhancement for TEM observation. The efficiency of silver enhancement is dramatically reduced by a prolonged illumination (28), making the use of fluoronanogolds difficult for CLEM analysis that requires extensive FLM observation time.

The second is to provide new tools to perform 3D analysis and correlation. Automated ETM, on both plastic sections and cryosections (29–32), allows us to obtain 3D images of cells with a resolution of about 3–8 nm, providing valuable information about the structural basis of many cellular processes. Tomograms are generated from the tilt series using a variety of software, for example IMOD(33) or iTEM (Olympus SIS). However, the examination of large complex structures requires surface rendering or volume rendering modeling and rotational tools to facilitate interpretation and measurements (30). This modeling requires a manual segmentation approach to define the limits of the selected structures, especially for cryosections (30). By this approach, the user traces the limits of the feature of interest with a mouse or a pen directly on the screen, repeating this operation for the several tens to hundreds of slices present in a tomography volume. Although informative, this procedure is time consuming and may lead to distortions because of manual drawing. In addition, for the most common electron microscope voltage set (i.e. 120–300 kV), tomography is limited to a section thickness of 200–400 nm.

However, in several instances, the analysis of a complex structure may require a better resolution compared with CLSM but not as extreme as in EM analysis. For instance, the 3D SRB convoluted structure could not be discriminated by a higher data output method such as CLSM (hundreds to thousands of events analyzed per single study section of a few hours) and required a very low data output and time-consuming ETM analysis (about a week of full-time work to generate a single model). By using 200 and 60 nm thick sections, there is a reduction in the out-of-focus background with respect to our discrimination abilities in the *x-y*plane so that the convoluted structure of the SRBs can be resolved already at the light microscopy level (compare Figure 1B with S4E). The use of thin sections allows a more precise quantitative evaluation of the amount of labeled macromolecules by means of fluorescence CLSM to be correlated with EM data. In addition ‘Turboreg MicroSCoBiOJ’, in combination with WAT MicroSCoBiOJ, helps speeding up the process of serial section alignment and modeling; a model of a complex structure such as a SRB can be obtained in a few hours. We have modeled RBs by the alignment of up to eight serial sections; therefore, a total thickness of about 1600 nm has been analyzed. In addition, ETM analysis from any of the serial sections composing a specific object can still be performed if higher resolution analysis is necessary. In this latter instance, the use of the fluorescence map allowed us to easily retrace the desired object in the serial sections.

The third is to allow a CLEM hybrid morphometry analysis, based on integrated multimodal data set that combines the high data analysis capability of FLM, with the high precision–accuracy of TEM. Morphometry at the TEM can be particularly time consuming, especially on cryosections because of the low image contrast that hampers the use of reliable segmentation software to automatically identify the compartment borders. A solution to this bias is offered by the finding that systematic errors between FLM and TEM can be identified for different types of sample (i.e. RRBs, n-SRBs and d-SRBs in our model) when comparing surface estimates obtained by CLEM analysis on the same structures by CLSM and TEM. Therefore, once a systematic error is identified by CLEM on a limited number of structures, it can be subsequently applied to the large-scale measurements obtained by CLSM to correct these values to the accuracy of the EM. Potentially, this method could also be applied to compare the labeling density of cell compartments by relating the fluorescence density obtained by CLSM analysis with the surface values obtained by HDO-CLEM morphometry.

The choice of SRB and RRB, as a complex and a simple model, respectively, was instrumental to the development of the HDO-CLEM method. Several aspects of the structure of these organelles were already previously defined by an EM analysis (17). HDO-CLEM did not add on to this knowledge for RRBs, and the added value was limited to the possibility to perform morphometry on a large number of organelles by the use of CSLM corrected for the systematic error. However, the understanding of the SRB morphology was improved by HDO-CLEM that helped define the 3D structure of a large number of organelles, at a resolution intermediate between the current CLSM analysis and the EM tomography, and set conditions for further studies. In particular, we are currently using this approach to study the potential connections between the RER and the SRB, as a way to unravel potential RER/ERGIC interactions (Valetti C, Tacchetti C. Centro di Ricerca MicroSCoBiO, Dipartimento di Medicina Sperimentale, Università di Genova, 16132, Genoa, Italy, personal communication). This type of studies must rely on a statistically significant large body of data provided by CLSM, by EM resolution to clearly identify the connections and by a fast 3D analysis to visualize connections that would be lost in a 2D analysis; HDO-CLEM fulfills these requirements.

## Materials and Methods

### Cell culture

Tet-Off HeLa cells were transfected with pTRE-CH1 (50 μg) and pTK-Hyg (5 μg; Clontech Laboratories), as described (17). Clones were grown in the presence of 3 μg/mL tetracycline, 400 μg/mL hygromycin and 100 μg/mL geneticin. Hygromycin-resistant clones were screened by immunofluorescence 72 h after tetracycline removal.

### CLEM specimen preparation

Cells fixed in 2% paraformaldehyde (Sigma-Aldrich) and 0.2% glutaraldehyde (PolyScience, Inc.) in PBS, pH 7.4, for 2 h were gently scraped-off the dish, embedded in 12% gelatin in PBS, cut into squared pieces and infiltrated with 2.3 m sucrose (Sigma-Aldrich) overnight at 4°C, mounted on pins, frozen in liquid nitrogen and sectioned at −100°C (200 nm cryosections) or −120°C (60 nm cryosections) with a Leica EM FCS Ultracut Microtome (Leica Microsystems). Flat ribbons of 60 or 200 nm thick cryosections, collected with 1.15 m sucrose and 1% 25 centipoise (cP) methylcellulose (Sigma-Aldrich), were transferred on formvar-coated 100-μm mesh, hexagonal gold finder grids (Agar Assing). For immunolabeling, samples were incubated on 2% gelatin in PBS for 20 min at 37°C, incubated with either rabbit anti-μ chain (Zymed) or rabbit anti-calreticulin (Stressgen) antibodies in 1% BSA in PBS for 30 min, rinsed in 1% BSA in PBS and incubated with donkey anti-rabbit Cy3-conjugated antibodies (Zymed) for 45 min in the presence of 4′,6-diamidino-2-phenylindole (DAPI) (Sigma) and PAG (10 nm) for 20 min (Department of Cell Biology, University Medical Center, Utrecht). For double labeling, after calreticulin labeling, samples were fixed for 5 min with 1% glutaraldehyde in PBS, washed in PBS and incubated with rabbit antibodies anti-μ chain (Zymed) for 30 min, rinsed in 1% BSA in PBS, incubated 45 min with donkey anti-rabbit Cy2-conjugated antibody (Zymed) in the presence of DAPI (Sigma), followed by PAG (15 nm) for 20 min, fixed for 2 min with 1% glutaraldehyde in PBS and washed in water. Unless otherwise indicated, all steps were at room temperature. For FLM observation, grids layered with a 200 nm coat of 2% methylcellulose were mounted between microscope slide and coverslip with 50% glycerol in water. For TEM observation, grids were unmounted, washed in water, incubated for 5 (200 nm cryosections) or 10 min (60 nm cryosections) in 2% uranyl acetate and 0.15 m oxalic acid and 5 min in 0.4% uranyl acetate and 1.8% 25ctp methylcellulose to increase contrast.

### Microscopy

CLSM fluorescence images were collected with an Olympus FV1000 spectral CLSM, equipped with a multiline Ar laser (100 mW, 457–476–488–514 nm), an HeNe(G) laser (1 mW, 543 nm) and an ultraviolet laser diode (405 nm). Images were acquired with a PlanApo ×60/1.42 oil objective (Olympus Europa GMBH). DAPI was excited at the absorption tails using the 405 nm laser diode, and fluorescence was collected in the 425–475 nm spectral region. Cy2 was excited at 488 nm, and fluorescence was collected in the 500–540 nm spectral region. Cy3 was excited at 543 nm, and fluorescence was collected in the 555–655 nm spectral region. Transmission imaging was obtained using a 543 nm laser line source. WFM fluorescence images were collected with an Olympus IX70 equipped with a 100 Watt mercury arc lamp using a low-magnification UPlanFL ×20/0.50 objective (Olympus Europa GMBH) or a high-magnification PlanApo ×100/1.40 oil objective (Olympus Europa GMBH). DAPI Ex 360–385 Em BA420, FITC Ex 470–490 Em BA515 Ex and TRITC Ex 510–550 Em BA590 filter sets were used to collect DAPI, CY2 and CY3 signal, respectively. Images were recorded with a digital C4742-95 Hamamatsu charge-coupled device (CCD) camera. Occasionally (e.g. GFP imaging), the Nikon C1si CLSM was used. TEM and ETM were performed on a Tecnai 12-G2 EM (FEI Company), equipped with Lab6 filament and compustage. Planar images and Panorama Image Function maps were recorded with a SIS MegaView III CCD camera (Olympus-SIS) using the iTEM (Olympus-SIS) software. Photomicrographs were obtained on Kodak Film 4489.

### Image processing and visualization

MicroSCoBiOJ was designed as a software toolbox collecting open source plug-ins for ImageJ(26). Some of the utilized plug-ins were optimized starting from exiting ones, and others were developed as original plug-ins. MosaicJ(23) assembles a mosaic of overlapping individual images and was used unmodified for high-resolution montages. Turboreg MicroSCoBiOJ, registers fluorescent physical section stacks, was modified from Turboreg to perform registration of serial multichannel images. WAT MicroSCoBiOJ, used for automatic surface area estimation on CLSM RB images, was fully developed in our laboratory using a segmentation algorithm based on a combined statically and dynamic threshold (see Segmentation in File S1). Basic functions of ImageJ were used for manual surface area estimation on TEM RB images. To obtain overlays of CLEM images, the pixel size of a FLM image was rescaled to that of the EM image and registered using the ‘rigid body rotation’ function of Turboreg (25) to avoid rotational differences. Merge of aligned/registered multichannel FLM image and grayscale TEM image was performed using Image5D plug-in. 3D fluorescence volume rendering was obtained combining two plug-ins developed in our laboratory: ‘Mesh Maker MicroSCoBiOJ’ implemented the Marching Cube (34) algorithm to extract isosurfaces from the 3D fluorescence volume data (see Surface rendering in File S1) and ‘Mesh Viewer MicroSCoBiOJ’ used the Java3D library (35) to render the set of the extracted isosurface. Tomograms of the tilt series were generated using the IMOD program package (33) or iTEM (Olympus SIS) software. PAG was used as fiducial marker.

## Supporting Information

File S1 Word document containing supplementary Material and Methods, Figures S1–S6, Tables S1–S2 and legends of Videos S1–S10.

Video S1 Tilt series (−45° to +45°, with 1° increments) of RRBs shown in Figure 1K.

Video S2 Demo of ‘Mesh_Viewer_MicroSCoBiOJ’ showing the surface rendering of RRBs and chromosomes shown in Figure 3G.

Video S3 Demo of ‘Mesh_Viewer_MicroSCoBiOJ’ showing the surface rendering of the SRB and nuclei shown in Figure 3H.

Video S4 Surface rendering of RRBs model shown in Figure 4J. Virtual sections of the tomogram are set as background images.

Video S5 Surface rendering of d-SRB model shown in Figure 4K. Virtual sections of the tomogram are set as background images.

Video S6 Surface rendering of n-SRB model shown in Figure 4L. Virtual sections of the tomogram are set as background images.

Video S7 Surface rendering of RRB model shown in Figure 4J.

Video S8 Surface rendering of d-SRB model shown in Figure 4K.

Video S9 Surface rendering of n-SRB model shown in Figure 4L.

Video S10 Demo of ‘Mesh_Viewer_MicroSCoBiOJ’ showing the surface rendering of the d-SRB model shown in Figure S4E.

Figure S1 CLEM approach applied to endogenously expressed Golgi and endosome antigens on 60 nm sections A–P) Sixty-nanometer cryosections of HeLa cells. A and B) Immunofluorescence labeling for Giantin (red, rabbit anti Giantin kind gift from Dr Antonella De Matteis, Mario Negri Sud and S. Maria Inbaro, Italy), a marker for Golgi, revealed with second Cy3-labeled antibodies. Nuclei are stained with DAPI (blue). C and D) Immunofluorescence labeling for LAMP-1 (red, H4A3, mouse anti-human LAMP1 monoclonal antibody obtained from the Developmental Studies Hybridoma Bank, Department of Biological Sciences, University of Iowa, Iowa City, IA, USA), a marker for late endosomes/lysosomes, revealed with second Cy3-labeled antibodies. Nuclei were stained with DAPI (blue). WFM images were collected with a with an Olympus IX70 equipped with a 100 Watt mercury arc lamp using a low-magnification UPlanFL ×20/0.50 objective (Olympus Europa GMBH) or a high-magnification PlanApo ×100/1.40 oil objective (Olympus Europa GMBH). E–H) Low-magnification EM of (A) and areas boxed in (B–D), respectively. I–L) Medium-magnification EM images of area boxed in (E–H), respectively. M–P) High-magnification EM images of area boxed in (I–L), respectively. Scale bars: 2.8 μm (A), 7.3 μm (B–D), 2.1 μm (E), 3.8 μm (F and G), 4.0 μm (H), 1.6 μm (I–L) and 0.8 μm (M–P).

Figure S2 A) Higher magnification and larger area of view of EM picture shown in Figure 1F. The image has been rotated 90° CW to better fit the page format. Bar: 1.1 μm. B) Higher magnification and larger area of view of EM picture shown in Figure 1G. The image has been rotated 90° CW to better fit the page format. Bar: 1.1 μm.

Figure S3 Model for resolution power comparison between CLSM optical sectioning and CLSM physical sectioning A) Simulation of CLSM optical sectioning imaging of a 3D phantom. Simulation was performed using the CLSM image acquisition conditions as for Figure 2K–M (i.e. 1.4 NA oil objective, 543 nm excitation wavelength, 565 nm emission wavelength and Pinhole Airy 1) (see Image process formation in File S1). The 3D phantom (red) represents a complex tubular structure (tubular width: ∼100 nm, tubular length: ∼1 μm). The phantom displays an area (white solid box) containing a fine branch within a *z*-thickness of 200 nm and an area (white dashed box) containing a large branch within a *z*-thickness of 400 nm. Surface rendering shows that it is not possible to separate the two branches of the phantom, when imaged by CLSM, because of limited axial resolution (blue halo). B) Simulation of CLSM imaging of 200 nm serial physical sections (cut along *z*-axis). As the axial resolution is increased, surface rendering (green halo) can now solve the large, but not the fine, branch of the phantom. This is principally because of the reduction of the background because of the partial removal of out-of-focus light. C) Middle section (*z* = 0) of a stack of CLSM optical sections through the phantom (blue halo) in (A). Blurring and out-of-focus light makes it impossible to solve tubular structures both in the fine and in the large branches. D) Middle section of the CLSM imaging of a stack of physical sections cut through the phantom (green halo) in (B). As out-of-focus light is partially reduced, now, it is possible to solve the large but not the fine branch. E) TEM image simulation for a 200 nm physical section cut through the middle of the phantom (*z* = 0). The 2D TEM image is not able to fully solve the fine branch because the large depth of focus superimposes features from different levels of the tubular structures. F) Simulation of ETM imaging for the central 200 nm physical section of the stack. Thanks to tomography, it is possible to obtain virtual slices at the nanometer resolution. Both fine and large branches are solved.

Figure S4 Application of FLM 3D reconstruction from physical sections reveals a hidden tubular architecture A) CLSM imaging of a single cryosection through a SRB (Figure 2L). Physical sectioning does not allow the identification of tubular connections in planes above and below a given section. 3D modeling of multiple serial sections (B and D) can solve this limitation. Three apparently separated areas [blue, yellow and magenta dots in (A)], identified in the SRB during the 2D analysis, are found to be connected to the adjacent tubular network by 3D modeling (E) but not by single-section analysis. E) Surface rendering obtained from the ROI sequence (B and D). Colored dots refer to the corresponding dots in (A) (Video S10).

Figure S5 CLEM approach applied to recombinantly expressed GFP–Rab5 A–H) Two hundred nanometer cryosections of HeLa cells transiently transfected with Rab5–GFP (kindly provided by Giorgio Scita, IFOM). A–C) Rab5–GFP localizes in discrete compartments (green) of a transfected cell. Images refer to the same ROI in three consecutive serial sections. Nuclei are stained by DAPI (blue). FLM images were collected with a Nikon C1si spectral CLSM, equipped with a multiline Ar laser (40 mW, 488/514 nm) and an ultraviolet laser diode (17 mW, 408 nm) and with a CFI Plan Apochromat VC ×60/1.4 oil objective (Nikon Instruments Europe B.V.). DAPI was excited at the absorption tails using the 408 nm laser diode, and fluorescence was collected in the 425–475 nm spectral region. GFP was excited at 488 nm, and fluorescence was collected in the 500–540 nm spectral region. D) 3D surface rendering was obtained as described in Figure 3. E) Low-magnification TEM map of the mesh area containing the ROI displayed in (A). F) CLEM overlay of the area boxed in (E) and the CLSM image shown in (A). G) Enlarged TEM image of the area boxed in (F). Immunogold labeling using rabbit antibodies to GFP (Molecular Probe) and PAG (10 nm) identifies Rab5–GFP associated to the surrounding membranes. Inset represents a high-magnification TEM image of the boxed ROI. The GFP-positive compartment is morphologically identifiable as an endosome. H) 3D model of the boxed area in (G). ETM was obtained from a −45° to +45° tilt series, with 1° increments. Tilt series were used to obtain tomograms of 1024 × 1024 × 31 voxels with 1.5 × 1.5 × 1.5 nm voxel size. Computer-assisted tracing of the membrane contours on the tomogram was used to generate the 3D model of the endosome in (G). Moreover, to validate the possibility of EM to identify also unlabeled structures, we generated also the 3D model of a mitochondria (blue), located close to the endosome. Scale bars: 5 μm (A–C), 50 μm (E), 5 μm (F) and 1 μm (G).

Figure S6 Behavior of mean relative error for different combination of threshold weights

Table S1 Choice of method based on desired resolution and sample thickness.

Table S2 Time-table for HDO-CLEM analysis.
