# Supplementary material for: High Data Output and Automated 3D Correlative Light–Electron Microscopy Method
Source: Traffic. 2008 Sep 16;9(11):1828–38. doi: 10.1111/j.1600-0854.2008.00815.x (PMC2635477; doi:10.1111/j.1600-0854.2008.00815.x)
Supplement: File S1 — Word document containing supplementary Material and Methods, Figures S1–S6, Tables S1–S2 and legends of Videos S1–S10. [file tra0009-1828-SD1.doc]

**High Data Output and Automated 3D Correlative Light-Electron Microscopy Method**

Vicidomini et al.

**Supplementary material**

***Content*:**

- Supplementary Material and Methods
- Supplementary Figures 1-6
- Supplementary Tables 1-2
- Supplementary Video legends 1 - 10

Supplementary Materials and Methods

*Surface rendering*

Surface rendering is a method used to display a 2D projection of an iso-surface representing a 3D data set. The first step for surface rendering is the extraction of the surface (iso-surface) at the boundary between background and specific labeling, from the 3D fluorescence sampled data set. The iso‑surface is usually approximated by a set of polygonal meshes. Lorensen and Cline(1) developed one of the most widely used algorithms for surface rendering, called Marching Cubes (MC) algorithm. The basic principle behind the MC algorithm is to subdivide the 3D data set into a series of small cubes, each one defined by its vertices and by the corresponding grey-scale intensity values. Using a specific iso-value it is possible to decide if a given vertex is inside or outside the iso-surface (i.e. the vertex is inside if its grey-scale intensity value is greater than the iso-value, otherwise it is ouside). If all vertex-values of a cube are greater, or lower, than the iso-value, than the iso‑surface does not pass through that cube. If the iso-surface passes through the cube, the MC algorithm creates a triangular patch that separates the cubes in the regions within the iso-surface, from those without. By connecting the patches obtained from all cubes on the iso-surface boundary, it is possible to get an approximation of the surface representation of the structure. The triangles obtained by the MC algorithm are then projected along the viewed direction to obtain the 2D surface rendering image. As reported by Düurst (2), the original version of the MC could determine holes in the iso-surface. In order to avoid this artefact we implemented a modified version of MC called MC reverse (3). In our implementation of the MC reverse the iso-value can be selected manually by the user, or automatically using the IsoData (4) algorithm. Future work will be directed to the definition of the best parameters to select the correct iso-value. We believe that an adaptive approach, as presented below, should lead to more realistic surface rendering.

***Segmentation***

Segmentation methods allow the identification of a structure of interest from a background area. There is no universally applicable segmentation method that will work for all images, and no segmentation technique is perfect. For this work we used a basic approach, implementing a segmentation algorithm based on two very common segmentation methods. However, more sophisticated algorithms could lead to more accurate results. The algorithm we used is based on the concept of threshold. A threshold value is computed for each pixel of an object image. If the pixel intensity is higher than the threshold, the pixel belong to the object. IsoData (4) algorithm identifies a global threshold for all pixels of an image, using statistical properties of the image histogram. Unfortunately this algorithm is not able to accommodate changes in the distribution of different fluorescence concentration in a structure. Chow and Kaneko (5) solved this problem dynamically changing the threshold over the image: each local pixel threshold is obtained by statistical investigation of the intensity values of the local neighbourhoods of such pixel. The statistic that is most appropriate depends largely on the input image: the more complex is the statistic, the better the results. For this work we combined the IsoData threshold (Th_IsoD_), with the dynamic threshold (Th_d_), to find a weight adaptive threshold (Th_wa_):

Th_base_ can be used to increase or decrease the final threshold on the base of prior information; moreover, further information, e.g. background, can be directly added to the algorithm. Supplementary Figure 4 shows the behavior of the mean relative error analysis on real confocal images for different combinations of the weights. It is evident that an accurate surface estimation can require a different combination of weights for different kinds of structure. For this reason the mean relative error analysis of Figure 5d both for real data were obtained with the following combination of weights, respectively, w_IsoD_ =0.0, w_d_ = 0.9, w_base_ = 0.1 (Th_base_=128) , b = 0 for RRB, w_IsoD_ =0.0, w_d_ = 1, w_base_ = 0.0, b = 0 for d-RRB, and w_IsoD_ =0.0, w_d_ = 1, w_base_ = 0.0, b = 0 for n-RRB. T_hd_ was computed using 12×12 pixels window. Before applied segmentation algorithm to real CLSM images a Gaussian blur filter (σ = 4pixel) was used to reduce noise. A sigma equal to 4 pixels ensured that only noise frequencies were cut, this value was more than two times lower than the FWHM of the PSF. It is noteworthy that the mean error on simulated data is lower than on real data, since the latter are corrupted by inevitable noise, and as expected performance of segmentation algorithms works better on free-noise images.

***Image process formation***

Image process formation in fluorescence microscopy can be modeled mathematically by the convolution between the function *f*, describing the concentration of fluorescence molecules in the specimen, and the point spread function (PSF) *k*, that describes the response of the system to a point-like source:

where *g* represents the measured intensity image. Note that *g*, *f* and *k* can be 3D or 2D functions, depending on the circumstances. For example, to simulate CLSM image, starting from manually segmented TEM image (Fig. 5), we used 2D functions, whereas to simulate the CLSM image formation of a tubular-structure (Supplementary Fig. 2) we used 3D functions. CLSM image simulation formation process was performed using a well-know PSF (6) model based on the vectorial (7) theory of light, able to take into account all the principal condition of CLSM imaging (NA and type of the objective, excitation and emission wavelength, pinhole size).

**References in Supplementary Materials and Methods**

1. William EL, Harvey EC. Marching cubes: A high resolution 3D surface construction algorithm Proceedings of the 14th annual conference on Computer graphics and interactive techniques 1987: ACM Press; 1987. p. 163-169

2. Duurst MJ. Additional reference to marching cubes. Computer Graphics 1988;22(2):72-73.

3. Rajon DA, Bolch WE. Marching cube algorithm: review and trilinear interpolation adaptation for image-based dosimetric models. . Computerized Medical Imaging and Graphics, 2003;27(5):411-435.

4. Ridler T, Calvard S. Picture thresholding using an iterative selection method. IEEE Trans Systems Man Cybernet 1978 8:629-632.

5. Chow CK, T. K. Automatic Boundary Detection of the Left Ventricle from Cineangiograms. Comp Biomed Res 1972;5:338-410.

6. Bertero M, Boccacci P, Brakenhoff GJ, Malfanti F, Van der Voort HTM. Three-dimensional image restoration and super-resolution in fluorescence confocal microscopy. Journal of Microscopy 1990;157(1):3-20.

7. Boivin A, Wolf E. Electromagnetic Field in the Neighborhood of the Focus of a Coherent Beam. Phys Rev 1965;138(6B):B1561-B1565.


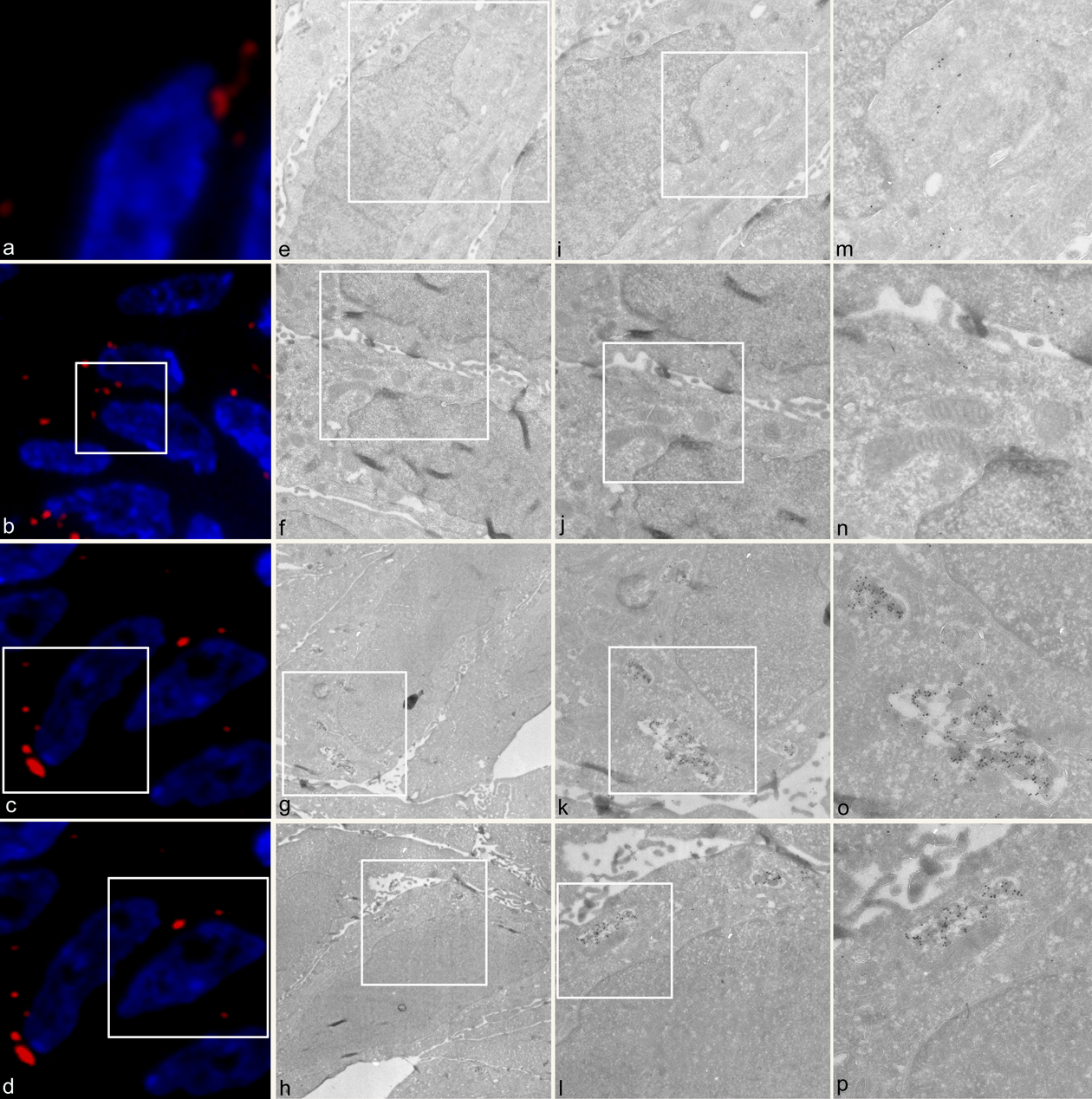
***Supplementary Figure 1. CLEM approach applied to endogenously expressed Golgi and endosome antigens on 60 nm sections.***

(**a**-**p**) 60 nm cryosections of HeLa cells. (**a**-**b**) Immunofluorescence labeling for Giantin (red, rabbit anti kind gift from Dr. Antonella De Matteis, Mario Negri Sud, S. Maria Inbaro, Italy), a marker for Golgi, revealed with second Cy3-labeled antibodies. Nuclei are stained with DAPI (blue). (**c**-**d**) Immunofluorescence labeling for LAMP-1 (red, H4A3, mouse anti-human LAMP1 monoclonal antibody obtained from the Developmental Studies Hybridoma Bank, University of Iowa, Dept of Biological Sciences, Iowa City, IA), a marker for late endosomes/lysosomes, revealed with second Cy3-labeled antibodies. Nuclei were stained with DAPI (blue). WFM images were collected with a with an Olympus IX70 equipped with a 100 watt mercury arc lamp, using a low-magnification UPlanFL 20X/0.50 objective (Olympus Europa GMBH), or a high-magnification PlanApo 100X /1.40 oil objective (Olympus Europa GMBH). (**e-h**) Low magnification EM of **a** and areas boxed in **b-d**, respectively. (**i-l**) Medium magnification EM images of area boxed in **e-h**, respectively. (**m-p**) High magnification EM images of area boxed in **i-l**, respectively. Scale bars: 2.8 μm (**a**), 7.3 μm (**b-d**), 2.1 μm (**e**), 3.8 μm (**f-g**), 4.0 μm (**h**), 1.6 μm (**i-l**), 0.8 μm (**m-p**).


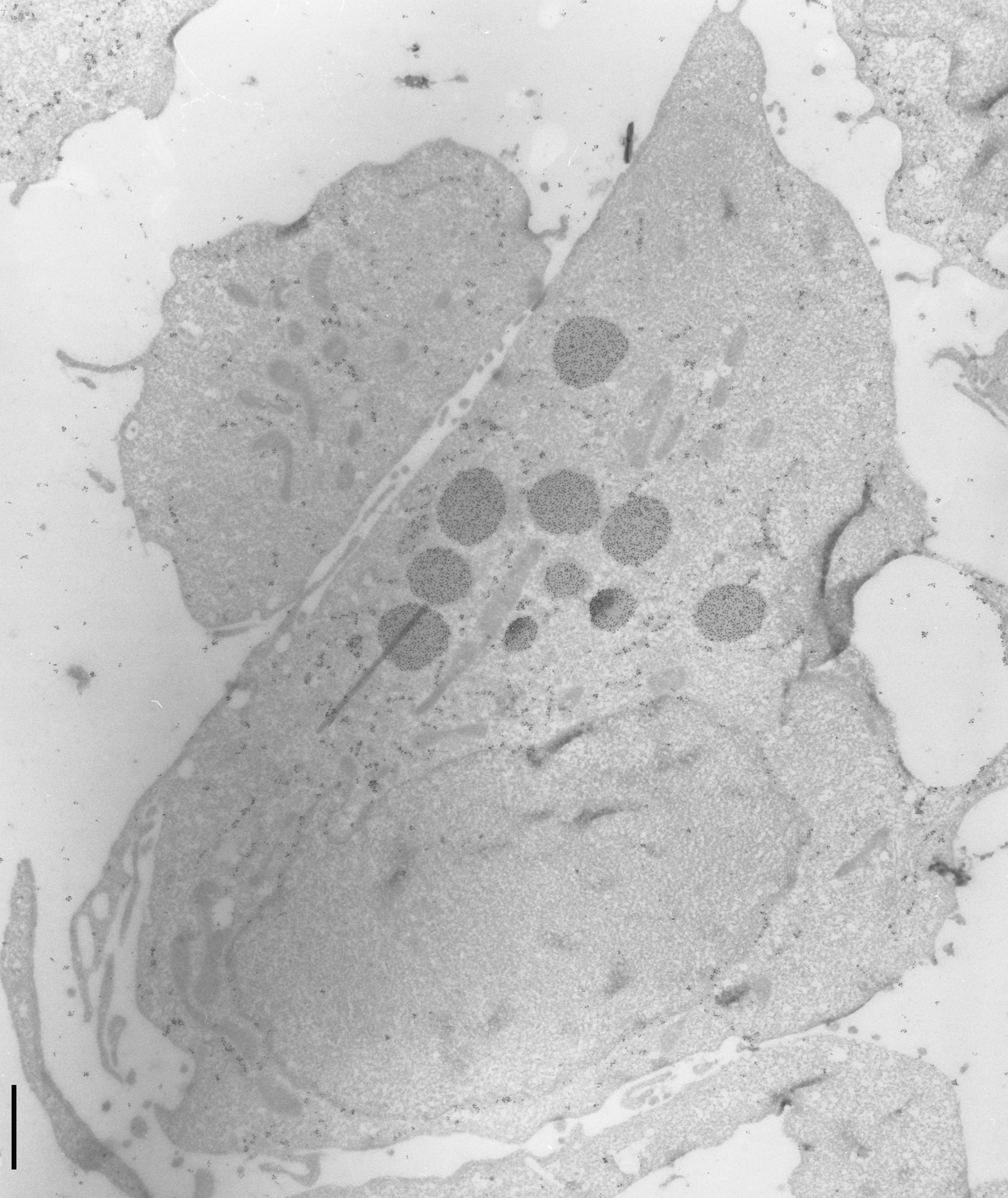


***Supplementary Figure 2a. Higher magnification and larger area of view of EM picture shown in Figure 1f.*** The image has been rotated 90° CW to better fit the page format. Bar: 1.1 µm.

***
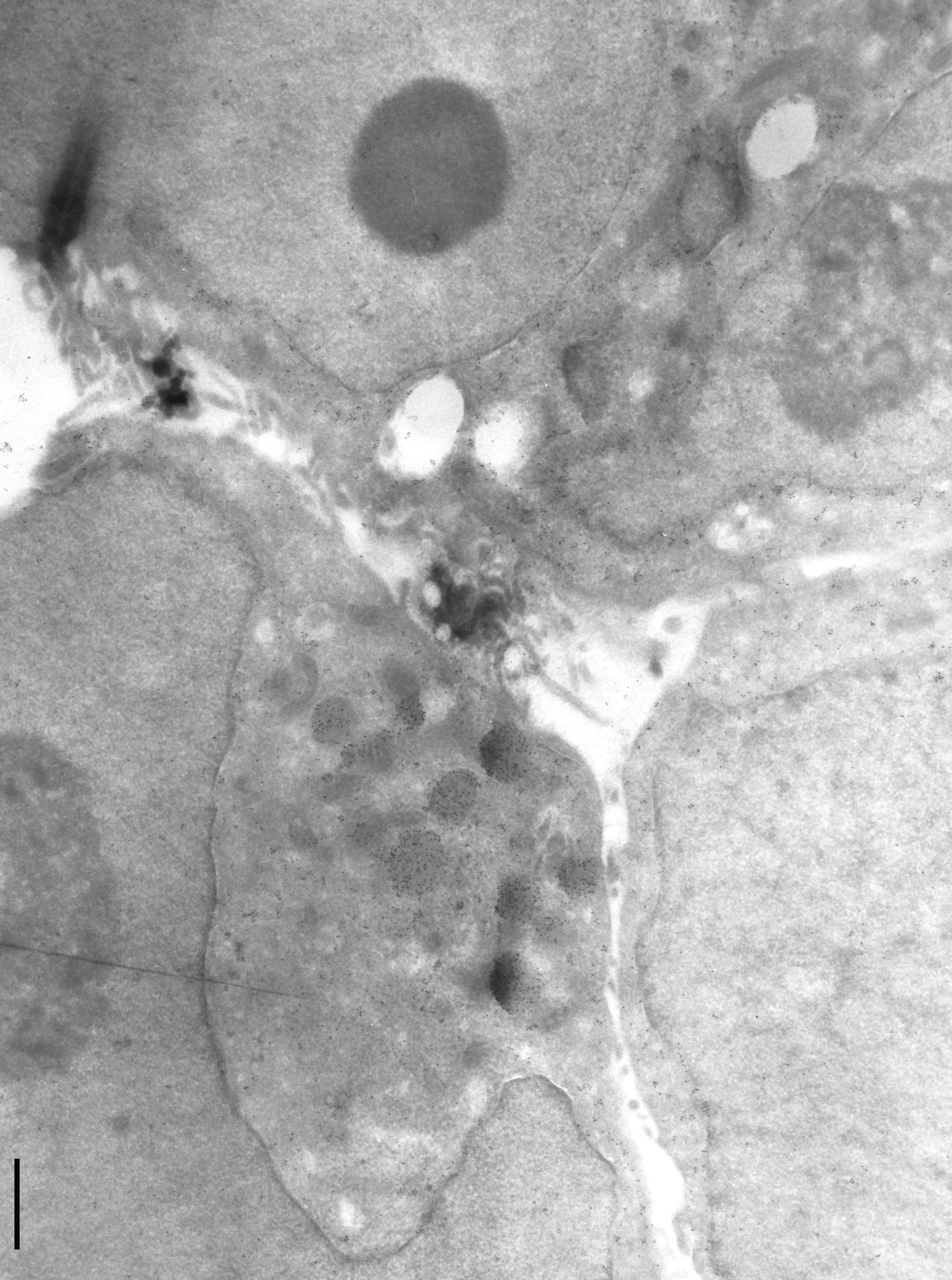
***

***Supplementary Figure 2b. Higher magnification and larger area of view of EM picture shown in Figure 1g.*** The image has been rotated 90° CW to better fit the page format. Bar: 1.1 µm.

**
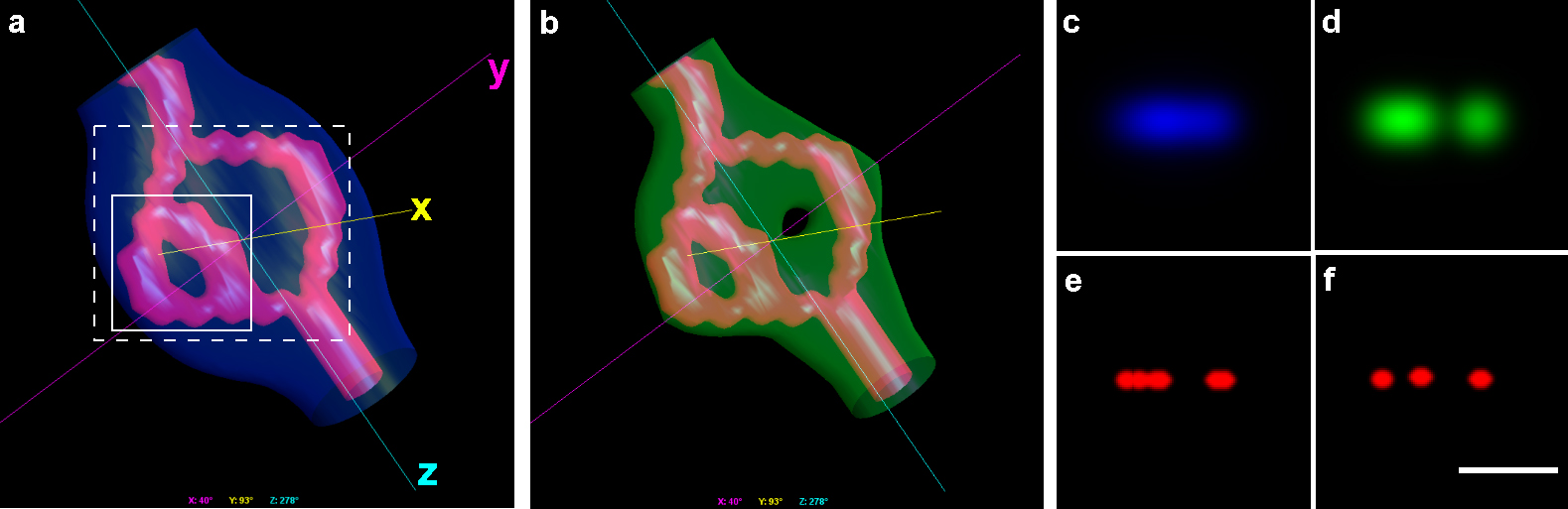
**

***Supplementary Figure 3. Model for resolution power comparison between CLSM optical‑sectioning, and CLSM physical-sectioning.***

(**a**) Simulation of CLSM optical-sectioning imaging of a 3D phantom. Simulation was performed using the CLSM image acquisition conditions as for figure 2k-m (i.e. 1.4 NA oil objective, 543 nm excitation wavelength, 565 nm emission wavelength, and Pinhole Airy 1)(Supplementary Materials). The 3D phantom (red) represents a complex tubular structure (tubular width: ~100 nm, tubular length: ~1 μm). The phantom displays an area (white solid box) containing a fine branch, within a z-thickness of 200 nm, and an area (white dashed box) containing a large branch within a z-thickness of 400 nm. Surface rendering shows that it is not possible to separate the two branches of the phantom, when imaged by CLSM, due to limited axial resolution (blue halo). (**b**) Simulation of CLSM imaging of 200 nm serial physical-sections (cut along z-axis). As the axial‑resolution is increased, surface rendering (green halo) can now solve the large, but not the fine, branch of the phantom. This is principally due to the reduction of the background, because of the partial removal of out-of-focus light. (**c**) Middle section (z=0) of a stack of CLSM optical-sections through the phantom (blu halo) in (**a**). Blurring and out-of-focus light makes it impossible to solve tubular structures, both in the fine and in the large branches. (**d**) Middle section of the CLSM imaging of a stack of physical-sections cut through the phantom (green halo) in (**b**). As out-of-focus light is partially reduced, now it is possible to solve the large, but not the fine branch. (**e**) TEM image simulation for a 200 nm physical‑section cut through the middle of the phantom (z=0). The 2D TEM image is not able to fully solve the fine branch, because the large depth of focus superimposes features from different levels of the tubular structures. (**f**) Simulation of ETM imaging for the central 200 nm physical‑section of the stack. Thanks to tomography, it is possible to obtain virtual slices at the nanometre resolution. Both fine and large branches are solved.

***
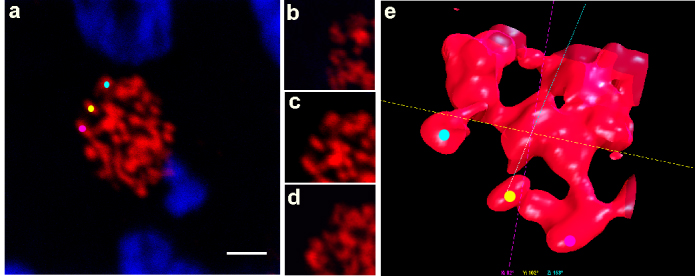
***

***Supplementary Figure 4. Application of FLM 3D reconstruction from physical‑sections reveals a hidden tubular architecture.***

(**a**) CLSM imaging of a single cryosection through a SRB (Fig. 2l). Physical-sectioning does not allow the identification of tubular connections in planes above and below a given section. 3D modelling of multiple serial sections (**b**-**d**) can solve this limitation. Three apparently separated areas (blue, yellow and magenta dots in **a**), identified in the SRB during the 2D analysis, are found to be connected to the adjacent tubular network by 3D modelling (**e**), but not by single section analysis. (**e**) Surface rendering obtained from the ROI sequence (**b**-**d**). Coloured dots refer to the corresponding dots in (**a**) (Supplementary Video 10).

***
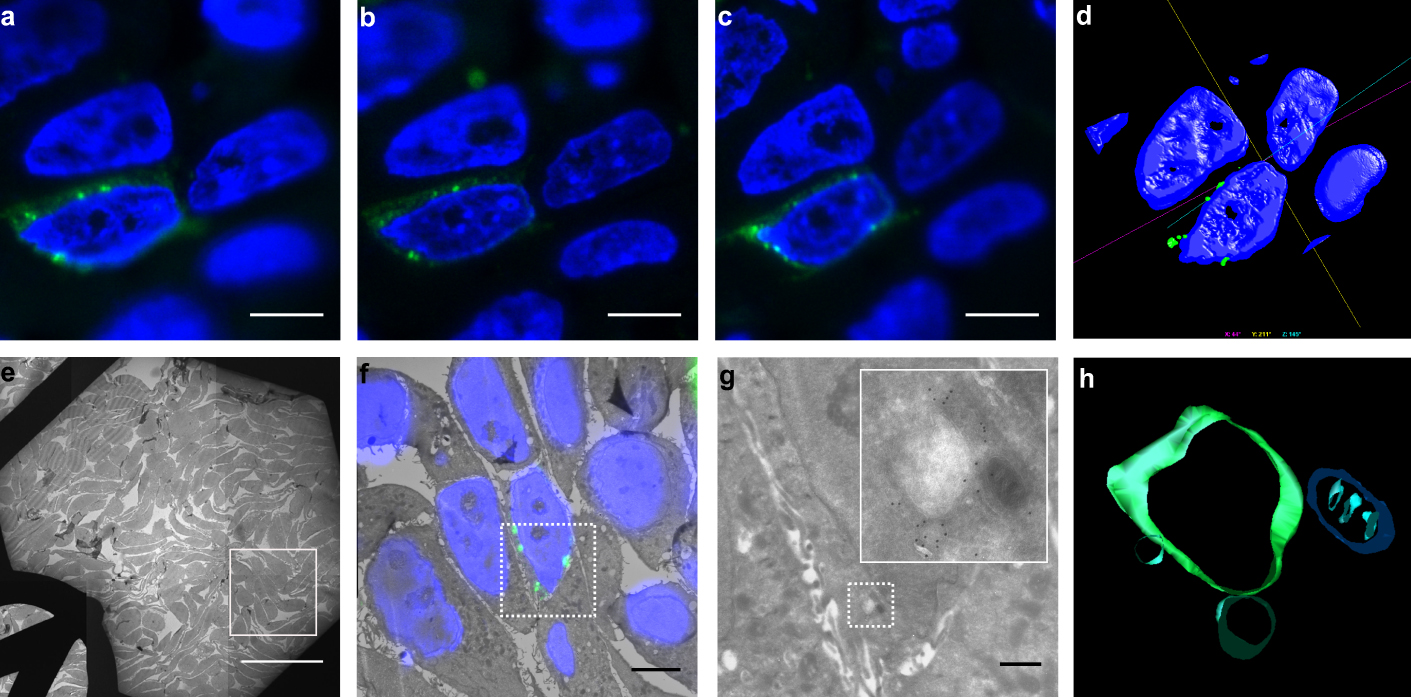
***

***Supplementary Figure 5. CLEM approach applied to recombinantly expressed GFP-Rab5.***

(**a**-**h**) 200 nm cryosections of HeLa cells transiently transfected with Rab5-GFP (kindly provided by Giorgio Scita, IFOM, Milan). (**a**-**c**) Rab5-GFP localizes in discrete compartments (green) of a transfected cell. Images refer to the same ROI in three consecutive serial sections. Nuclei are stained by DAPI (blue). FLM images were collected with a Nikon C1si spectral CLSM, equipped with a multi-line Ar laser (40 mW, 488/514 nm) and an UV laser diode (17 mW, 408 nm), and with a CFI Plan Apochromat VC 60X/1.4 oil objective (Nikon Instruments Europe B.V.). DAPI was excited at the absorption tails using the 408 nm laser diode and fluorescence was collected in the 425-475 nm spectral region. GFP was excited at 488 nm and fluorescence was collected in the 500-540 nm spectral region. (**d**) 3D surface rendering obtained as described in figure 3. (**e**) Low magnification TEM map of the mesh area containing the ROI displayed in (**a**). (**f**) CLEM overlay of the area boxed in (**e**), and the CLSM image shown in (**a**). (**g**) Enlarged TEM image of the area boxed in (**f**). Immunogold labeling, using rabbit antibodies to GFP (Molecular Probe) and PAG (10 nm), identifies Rab5-GFP associated to the surrounding membranes. Inset represents a high magnification TEM image of the boxed ROI. The GFP-positive compartment is morphologically identifiable as an endosome. (**h**) 3D model of the boxed area in (**g**). ETM was obtained from a -45° to +45° tilt series, with 1° increments. Tilt series were used to obtain tomograms of 1024 x 1024 x 31 voxels with 1.5 x 1.5 x 1.5 nm voxel size. Computer-assisted tracing of the membrane contours on the tomogram was used to generate the 3D model of the endosome in (**g**). Moreover, to validate the possibility of EM to identify also unlabeled structures, we generated also the 3D model of a mitochondria (blue), located close to the endosome. Scale bars: 5 μm (**a**-**c**), 50 μm (**e**), 5 μm (**f**), 1 μm (**g**).

***Supplementary Figure 6. Behavior of mean relative error for different combination of threshold weights.***

| **Technique** | **Lateral Resolution** | **Axial  Resolution** | **Sample thickness** |
| --- | --- | --- | --- |
| WFM | ~ 200 nm | Comparable to CLSM when combined with deconvolution (1) | Whole cell |
| CLSM | ~ 180 nm | ~ 500-800 nm | Whole cell |
| TEM | ~ 2-4 nm (2,3) | n/a  *(2D technique)* | n/a |
| ETM  *single axis*^a^ | d_x_ ≈ 2-4 nm  d_y_ ≈ 5 nm | d_z_ ≈ 8 nm | Thickness of the physical section |
| ETM  *dual axis*^b^ | d_x_ ≈ 2-4 nm  d_y_ ≈ 2-4 nm | d_z_  < 8 nm  *(*~ *Isotropic resolution)* | Thickness of the physical section |
| HDO-CLEM:  *CLSM* | ~ 140 nm | Thickness of cryo-section^d^ | Whole cell^c^ |
| HDO-CLEM: *TEM + CLSM* | ~ 2-4 nm | Thickness of cryo-section^d^ | Whole cell^c^ |
| HDO-CLEM:  *CLSM + ETM* | ~ 2-4 nm | d_z_  < 8 nm  *(*~ *Isotropic resolution)* | Whole cell^c^ |

**Supplementary table 1**. **Choice of method based on desired resolution and sample thickness.**

WFM (wide-field microscopy);   CLSM (confocal laser scanning microscopy); TEM (trasmission electron microscopy); ETM (electron tomography microscopy); HDO-CLEM (high data output-correlative light/EM microscopy); n/a (not applicable)

^a^In general, for the single-axis tilt geometry, the spatial resolution of the reconstruction is anisotropic. Parallel to the tilt axis, say the x-axis, the resolution, *d_x_*, is equal to the original resolution of the projections, assuming a perfect tilt series alignment. The resolution in the other perpendicular directions is controlled by the number of projections acquired, *N*, and the diameter, *D*, of the volume to be reconstructed and is given by the Crowther relation (4):

Hoverer for different reason, this criterion has a limited significance as a resolution measure for cellular tomography. First, for most application the molecular resolution is not limited by the Crowther criterion but by the structural preservation of specimen or the noise. Second Crowther criterion is derived for specimens that are circular and perpendicular to the tilt axis, but the specimens usually have slab geometry, and for a constant tilt increment between -90 to +90 degree, but this in practice not realizable. Nevertheless, this criterion remains an important means of interpreting tomograms.

Moreover, Crowther criterion can be corrected for limits in tilt range: in practice, the limited space between the objective lens pole pieces and the finite thickness of the specimen holder limits the tilt range, giving rise to the ‘missing wedge’ in Fourier space (5). This missing information leads to the resolution in the direction parallel to the optic axis, dz, being degraded further by an ‘elongation factor’, *e_yz_*, so that *d_z_ = d_y_e_yz_* which is related to the maximum tilt angle, *α* (in radians), by:

As a consequence, reconstructed objects appear elongated in the beam direction, and some structural elements are not resolved at all. Using this formulation, the resolutions *d_y_* and *d_z_* for a topographic reconstruction of a specimen of 200 nm thickness are ~ 5 nm and ~ 8 nm, respectively, if the tilt angle interval is 1 and the maximal tilt angle is 60°.

^b^The acquisition of a double-tilt series can partially correct the elongation degradation; two single-tilt series of the objects are recorded during which the specimen is rotated by 90° around the beam direction after the first series. This acquisition procedure samples the structural factors of the objects more isotropically, so only a pyramid-shaped region in Fourier space remains unsampled. For example the dual-axis scheme for a maximal tilt angle of 60° increase from 67% to 93% the percentages of the Fourier space that is covered (5). Consequently, although the maximum resolution is not increased, the achievable resolution is more isotropic.

^c^ Fluorescence map obtained by HDO-CLEM technique thereby reduced the time necessary to scout the serial cryo-sections in order to find consecutive physical sections of the same cellular structure.

^d^Single images (TEM or CLSM) of individual physical sections of the same cellular structure are computationally merged to obtain a three-dimensional reconstruction, limiting the resolution in the z-direction to the slice thickness.

**References in Supplementary Table 1.**

1. P.J. Shaw, Comparison of wide-field/deconvolution and confocal microscopy for 3D imaging. In: JB Pawley, Editor, (edn 2 ed.), Handbook of Biological Confocal Microscopy, Plenum, New York (1995), pp. 373–387. In: Pawley JB, editor. Handbook of biological confocal microscopy. Second ed. New York: Springer; 2006

2. B.F. McEwen and M. Marko, The Emergence of Electron Tomography as an Important Tool for Investigating Cellular Ultrastructure, The Journal of Histochemistry & Cytochemistry, 2001, 49(5): 553–563.

3. A.J. Koster, R. Grimm, D. Typke, R. Hegerl, A. Stoschek, J. Walz, W. Baumeister, Perspectives of Molecular and Cellular Electron Tomography, Journal of Structural BiologyVolume, 1997, 120(3):276-308.

4. R.A. Crowtherde, D.J. Rosier, A. Klug, The reconstruction of a three-dimensional structure from projections and its application to electron microscopy. Proc. Roy. Soc. Lond. A, 1970, 317:319–340.

5. V. Lucic, F. Foerster, W. Baumeister, Structural studies by electron tomography: From cells to molecules, Ann. Rev. Biophys. Biomolec. Struct., 2005, 74(1):833–865.

|  | **2D analysis** | |  | **3D analysis** | |
| --- | --- | --- | --- | --- | --- |
|  | Fluorescence Microscopy (WFM/CLSM) | TEM |  | WFM/CLSM + modeling | ETM  + modeling |
| Single  immuno-labeling | 3 hrs | 4 hrs | Single axis | n/a | 6 hrs ** |
| Multiple  immuno-labeling | +2 hrs* | +3 hrs* | Double axis | n/a | 12 hr ** |
| Re-tracing + overlay | 1 hr ** | | Re-tracing + overlay | 4 hr *** | 7 hrs ** |

**Supplementary table 2**. **Time-table for HDO-CLEM analysis.**

Approximate timing of each relevant step for HDO-CLEM. 3D analysis requires a previous 2D analysis on the same sample.

+ additional to single labelling, for each additional marker.

** for each ROI;

***for the first two sections, and for each additional section after the first two

WFM (wide-field microscopy);   CLSM (confocal laser scanning microscopy); TEM (Transmission Electron Microscopy); ETM (Electron Tomography Microscopy); n/a (not applicable)

**Supplementary Video 1**. Tilt series (-45° to +45° with 1° increments) of RRBs shown in Figure 1k.

**Supplementary Video 2.** Demo of ‘Mesh_Viewer_MicroSCoBiOJ’ showing the surface rendering of RRBs and chromosomes shown in Figure 3g.

**Supplementary Video 3.** Demo of ‘Mesh_Viewer_MicroSCoBiOJ’ showing the surface rendering of the SRB and nuclei shown in Figure 3h.

**Supplementary Video 4.** Surface rendering of RRBs model shown in Figure 4j. Virtual sections of the tomogram are set as background images.

**Supplementary Video 5.** Surface rendering of d-SRB model shown in Figure 4k. Virtual sections of the tomogram are set as background images.

**Supplementary Video 6.** Surface rendering of n-SRB model shown in Figure 4l. Virtual sections of the tomogram are set as background images.

**Supplementary Video 7.** Surface rendering of RRB model shown in Figure 4j.

**Supplementary Video 8.** Surface rendering of d-SRB model shown in Figure 4k.

**Supplementary Video 9.** Surface rendering of n-SRB model shown in Figure 4l.

**Supplementary Video 10.** Demo of ‘Mesh_Viewer_MicroSCoBiOJ’ showing the surface rendering of the d-SRB model shown in Supplementary Figure 2e.
